# Association Between the Combined Herbal Medicines and Risk of Dental Diseases in Patients with Rheumatoid Arthritis: Insight from a Nationwide Database

**DOI:** 10.3390/medicina62040767

**Published:** 2026-04-15

**Authors:** Chiu-Hui Ling, Wei-Jen Chen, Ying-To Hsu, Hanoch Livneh, Ming-Chi Lu, Tzung-Yi Tsai

**Affiliations:** 1 Department of Dentistry, Dalin Tzu Chi Hospital, Buddhist Tzu Chi Medical Foundation, Chiayi 62247, Taiwan; e221672@tzuchi.com.tw; 2 Center of Sports Medicine, Dalin Tzu Chi Hospital, Buddhist Tzu Chi Medical Foundation, Chiayi 62247, Taiwan; tough2915@hotmail.com; 3 School of Post-Baccalaureate Chinese Medicine, Tzu Chi University, Hualien 97004, Taiwan; 4 Graduate Institute of Sports Science, National Taiwan Sport University, Taoyuan 333325, Taiwan; 5 Division of Allergy, Immunology and Rheumatology, Dalin Tzu Chi Hospital, Buddhist Tzu Chi Medical Foundation, Chiayi 62247, Taiwan; dl32399@tzuchi.com.tw; 6 Rehabilitation Counseling Program, Portland State University, Portland, OR 97207-0751, USA; livnehh@pdx.edu; 7 School of Medicine, Tzu Chi University, Hualien 97004, Taiwan; 8 Department of Medical Research, Dalin Tzu Chi Hospital, Buddhist Tzu Chi Medical Foundation, Chiayi 62247, Taiwan; 9 Department of Environmental and Occupational Health, College of Medicine, National Cheng Kung University, Tainan 70428, Taiwan

**Keywords:** rheumatoid arthritis, herbal medicines, dental diseases, cohort study, risk

## Abstract

*Background and Objectives:* Patients with rheumatoid arthritis (RA) are found to have a higher risk of dental diseases. Although herbal medicines (HMs) have long been used to treat various conditions, few studies focus on its impact on dental diseases. In this longitudinal cohort study, we assessed the correlation between HM use and risk of dental diseases in RA groups. *Materials and Methods:* A total of 2359 persons with RA aged 20–80 who were free of dental diseases between 2001 and 2010 were retrospectively enrolled from nationwide register-based data. They were then classified into HMs and non-HMs groups based on whether they ever used combined HMs after RA onset. Incidence rate and hazard ratios (HRs) of dental diseases were estimated for both groups by the end of 2013 via fitting Cox proportional hazards model. *Results:* Incidence rate of dental disease was reported to be lower in the HMs group than in the non-HMs group (90.21 per 1000 person-years versus 106.94 per 1000 person-years, respectively). RA individuals treated with HMs showed a significantly lower risk of dental diseases, especially dental caries, pulpitis, periodontitis, and stomatitis. Among commonly prescribed formulas, eleven herbal products significantly associated with a lower risk of dental diseases, such as Hai-Piao-Xiao, Yan-Hu-Suo, Chuan-Niu-Xi, Mo-Yao, Olibanum, Bei-Mu, Mu-Gua, Gui-Zhi-Shao-Yao-Zhi-Mu-Tang, Shao-Yao-Gan-Cao-Tang, Xue-Fu-Zhu-Yu-Tang, and Ping-Wei-San. *Conclusions:* The addition of HMs treatment may have advantages to proactively prevent sequent risk of dental disorders for persons with rheumatic diseases. A deeper exploration focusing on pharmacological action is needed to provide more reliable evidence for the improvement of susceptible individuals’ oral hygiene.

## 1. Introduction

Inflammatory diseases are a diverse group of disorders characterized by aberrant chronic inflammation that endangers the entire body. Rheumatoid arthritis (RA) is a chronic inflammatory disease that affects individual joints, causing cartilage loss and bone erosion which provokes significant functional disability. The global incidence of RA increased from approximately 7.9 million in 1990 to almost 18 million in 2021, representing an increase of 125% [1,2], thus posing a heavy economic burden on the healthcare system. In the United States, a study on the Medicare program estimated that the total societal cost of RA amounts to roughly 40 billion dollars each year, and the estimated medical expenses for one RA individual per admission approximately totals USD 20,919, which is three times the expenditure for individuals without RA [3].

Importantly, not only posing a heavy economic toll, RA is recognized to progress into other co-occurring conditions beyond just the joints, particularly dental conditions [4]. A 2018 study estimated that nearly four-fifths of RA patients had oral disorders [5]. Our previous work also depicted that RA patients had a higher frequency of dental visits and an elevated risk of developing dental disorders, including dental caries, pulpitis, gingivitis, periodontitis, and oral ulceration in Taiwan [6]. Recent studies have explored the molecular mechanisms underlying the association between RA and gum disease. An aberrant influx of microbiological communities, such as *Porphyromonas gingivalis*, *Preponema denticola*, *Prevotella intermedia*, and *Tannerella forsythia*, have been noted in the synovial fluid during the early phase of RA [7]; these bacteria all contribute to the development or exacerbation of periodontal diseases [2,8,9]. Additionally, the endogenous inflammatory mediators associated with RA may promote periodontium immune cells to express matrix metalloproteinases and the receptor activator of the nuclear factor kappa B ligand, both of which are involved in alveolar bone destruction [10]. These findings may methodically summarize current understanding and mechanism of the crosstalk between RA and dental conditions. Notably, the clinical manifestations, encompassing teeth loss and a wide range of temporomandibular diseases, may impair susceptible individuals’ social and psychological function [11]. A review manifested that persons with periodontal conditions tended to have suicidal thoughts [12]. To grapple with this dilemma, RA management should extend beyond current monotherapy to seek out possible complementary medicines for mitigating oral diseases.

Herbal medicines (HMs), a specialized therapy in traditional Chinese medicine, have recently emerged as a focal point in treating various human diseases due to their safe and minimal side effects. A preclinical study showed that plant-derived compounds with prebiotic activity greatly improve gut microbiome stability, ameliorating systemic inflammation and gut microbiota dysbiosis [13]. The potential of herbal products, particularly compounds within Mu-Gua and Olibanum, has been noted to minimize the likelihood of oral disorders because of prominent antioxidant, anti-inflammatory, and antibacterial properties [14,15]. Another herbal formula, Dang-Gui-Nian-Tong-Tang, has been shown to alleviate the symptoms of bone erosion and joint destruction as well as control mitochondrial apoptosis by regulating the abundance of *prevotella* [16]. It is noteworthy that the family *prevotellaceae* has been found to promptly pile up in the gut soon after RA emergence, thus possibly impairing gum hygiene [17]. Furthermore, seeing as the addition of HMs treatment may decrease the risk of developing dental diseases in patients with Sjögren’s Syndrome [18], we speculated that adding HMs into routine care may act as a prophylactic manner to prevent dental diseases from occurring for those with RA.

While the existing evidence may underpin the potential for HMs in managing oral manifestations, the link between use of combined HMs and subsequent risk of dental diseases in RA patients remains unverified. Accordingly, we conducted a population-based retrospective study in a cohort of patients with RA to compare risk of dental diseases between those using combined HMs treatment and those receiving Western medicine only, which would be a reference to help establish holistic care unique for RA persons.

## 2. Methods

### 2.1. Data Source

The data for this retrospective cohort study were sourced from the National Health Insurance Research Database. This database is a continuous, multistage sampling database managed by the Health Welfare Data Science Center of Taiwan. The deposited data included beneficiary information, such as sex, birth date, and physician billing records, for comprehensive outpatient and inpatient visits covered by the National Health Insurance program. At present, almost 99% of Taiwanese residents are registered in this program [19]. The identification numbers of both individuals and healthcare providers have been transformed using cryptographic algorithms. All medical diagnoses are documented according to the International Classification of Diseases, Ninth Revision, Clinical Modification (ICD-9-CM). Besides complying with the ethical principles of the Declaration of Helsinki, this work has been approved by the Institutional Review Board of the Buddhist Dalin Tzu Chi Hospital (No. B10004021-3).

### 2.2. Rationalization of Study Cohort

**Figure 1:**
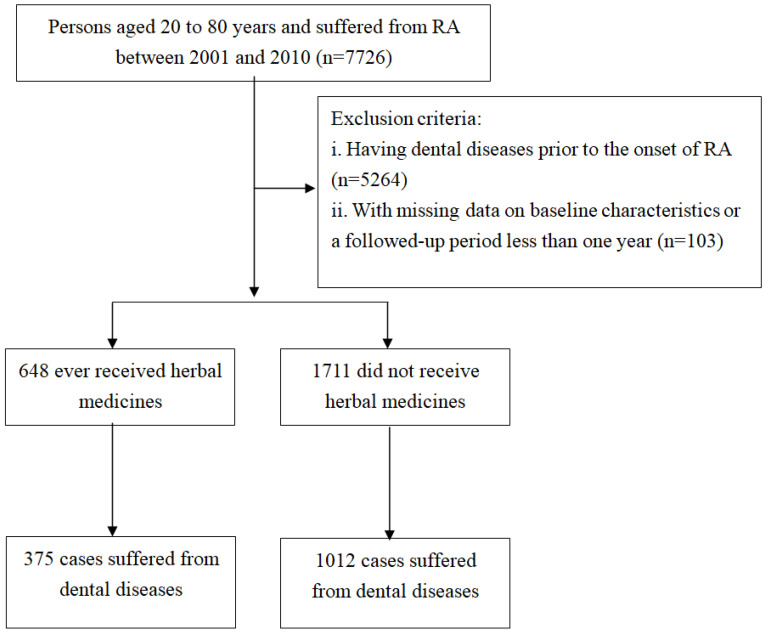
Flowchart of the recruited subjects.

The study population was restricted to those aged 20–80 years with RA between 2001 and 2010. Participants were classified as having RA upon at least one hospital admission or three or more outpatient visits marked with an ICD-9-CM code of 714.0, plus the prescription of corticosteroids or disease-modifying anti-rheumatic drugs for 6 months or more. These cases were further verified according to whether they held a catastrophic illness certificate. In Taiwan, people with specific diseases, such as mood disorders, autoimmune diseases or carcinoma, can apply for this certificate to gain compensation for each medical visit. Hence, the certificate approval date was regarded as the starting point for the time at risk of developing RA. We excluded 103 cases because their length of follow-up did not surpass one year or they had missing data on sex and age. Patients diagnosed with RA episodes after dental diseases were also excluded to ascertain the temporal direction (*n* = 5624). After applying these filters, 2359 patients with new-onset RA were included in this study (Figure 1).

### 2.3. Clarifications of Dental Diseases

Individuals were classed as suffering from dental diseases if they had at least three outpatient claims or at least one inpatient claim throughout the study frame that included the following codes: dental caries (ICD-9 code 521.0), pulpits (ICD-9 code 522.0), gingivitis (ICD-9 codes 523.0, 523.1 and 523.2), periodontitis (ICD-9 codes 523.3, 523.4, 523.5 and 523.8), oral ulceration (ICD-9 code 528.2), or stomatitis (ICD-9 code 528.0). All aforementioned indicators were restricted to those occurring after onset of RA; the date of the first diagnosis visit was used as the onset of the outcome of interest.

### 2.4. Exposure of HMs Use

After filtering, all participants were linked to ambulatory claims to verify HM use after the onset of RA. Under the National Health Insurance program, only practitioners with Chinese medicine licenses are qualified to prescribe HMs and provide relevant treatments. In accordance with a previously established method [20], the HMs group was defined as participants who had ever received HM treatment for RA along with conventional Western medicine for more than 30 days. To avoid misclassification of the unexposed period (immortal time) in the exposed group (HMs group), we calculated the person-years (PY) starting from the first day of HMs prescription to correct for immortal time. Conversely, the index date of the follow-up period for the non-HMs group was assigned as the date of the first RA diagnosis [21]. All enrollees were then followed until the end of 2013 for oral disease identification. The follow-up time in PY was determined by calculating the time interval from the index date to the following endpoints: diagnosis of dental disorders, termination of insurance coverage, or until the end of 2013, depending on which happened first.

### 2.5. Demographic Variables and Disease Characteristics

Demographic variables included in the analysis included age, sex, income for estimating insurance payments, and urbanization level of the participant’s residential area. The insured amount, calculated from the patients’ average monthly income, was chosen as a surrogate for the socioeconomic index and transformed into ordinal variables with predefined groups based on the 25th, 50th and 75th percentiles. We also grouped the region of each participant’s insurance unit by dividing the beneficiaries’ geographic locations into three regions (urban, suburban, and rural) according to an established formula, which considered the population density, the proportion of residents with at least a bachelor’s degree, ratio of inhabitants aged 65 years or older, percentage of the labor force in agriculture, and number of doctors per 100,000 residents [22]. This indicator enables us to assess the enrollees’ accessibility to healthcare services. With regard to disease characteristics, it referred to the illnesses occurring at least once in inpatient claims or twice in outpatient claims one year preceding cohort entry. Each disease was assessed using the Charlson–Deyo Comorbidity Index (CCI), a scoring system that measures 17 weighted comorbid conditions with higher scores indicating potentially greater impacts on health as a whole [23].

### 2.6. Statistical Analysis

Descriptive statistics of the patients’ characteristics were reported using mean ± standard deviation (SD) for continuous variables and frequencies for categorical variables. Differences in baseline characteristics between two cohorts were assessed using a two-sided *t*-test or χ^2^ test. Incidence rate of dental diseases was presented as the number of cases per 1000 PY. The Kaplan–Meier method was utilized to compare the cumulative incidence of dental diseases between two groups, and a log-rank test was applied to examine the significance level of differences between the two groups. Subsequently, Cox proportional hazards regression was applied to determine the association of HMs use with risk of dental disorders, shown as hazard ratios (HRs) and 95% confidence intervals (CIs). To test the robustness of this relationship, we divided the HMs group into three subgroups: Q1 (HMs use for less than one year), Q2 (HMs use for 1–2 years), and Q3 (HMs use for 2 years or more). Stratification by dental disease was also performed to assess the relative risk of different oral diseases in relation to uses of HMs. All analyses were conducted using SAS version 9.4 (SAS Institute Inc., Cary, NC, USA). A *p*-value below 0.05 was considered statistically significant.

## 3. Results

During the study period, 1711 participants merely received conventional Western medicine (non-HMs group) and 648 participants received conventional Western medicine combined with HMs (HMs group). Mean age for all patients was 57.3 years with a SD of 13.5 years. Females were the dominant sex in both groups. Collectively, no significant differences in baseline characteristics were detected between two cohorts, except for sex and CCI score. Said another way, compared to the non-HMs group, the HMs group was more likely to be female and have higher CCI scores (Table 1).

**Figure 2:**
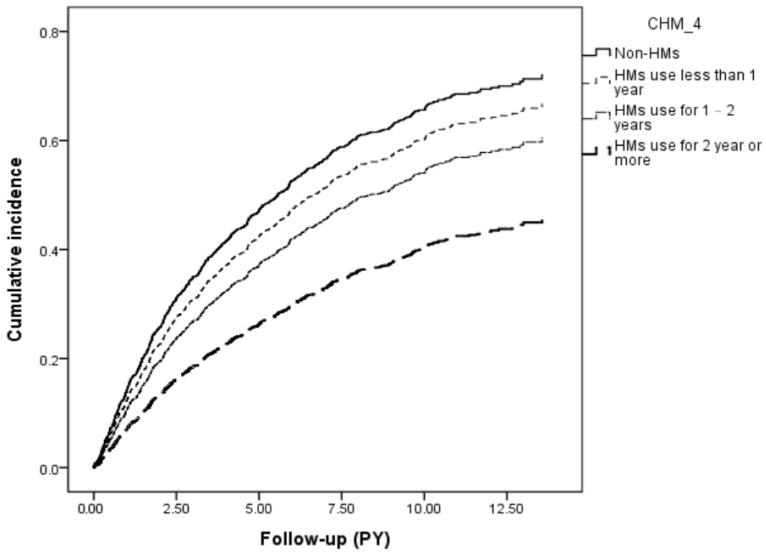
Cumulative incidence of dental diseases in RA patients with and without adding HMs therapy in Western medicine (Log-rank test, *p* < 0.001).

Among all participants, we observed 1387 first episodes of dental diseases, which comprised 1012 and 375 episodes in non-HMs and HMs groups, respectively, during follow-up periods of 9463.65 and 4156.79 PY. The incidence rate of dental diseases was lower in the HMs group than in the non-HMs group (90.21 vs. 106.94 per 1000 PY). According to the Cox proportional hazards regression model controlling for pertinent covariates, the HMs group exhibited a lower risk of dental diseases than the non-HMs group (adjusted HRs = 0.75; 95% CI: 0.66–0.86). Kaplan–Meier survival curve analysis and log-rank tests revealed a significant difference of incidence rate in dental diseases across the four groups (*p* < 0.001) (Figure 2). Subgroup analysis further depicted the frequency of HMs use significantly related to the likelihood of developing dental diseases, exhibiting an inverse exposure–response correlation, with the adjusted HRs of 0.81 (95% CI: 0.71–0.91), 0.48 (95% CI: 0.33–0.72), and 0.32 (95% CI: 0.15–0.68) for Q1, Q2 and Q3 separately (Table 2). Table 3 portrays the risk of developing different dental diseases according to HMs use. Generally, the combined HMs treatment could lower the risk of dental caries (adjusted HR 0.75; 95% CI: 0.61–0.94), pulpitis (adjusted HRs 0.65; 95% CI: 0.43–0.98), periodontitis (adjusted HRs 0.74; 95% CI: 0.60–0.91) and stomatitis (adjusted HRs 0.63; 95% CI: 0.42–0.94), but not for gingivitis and oral ulceration.

**Figure 3:**
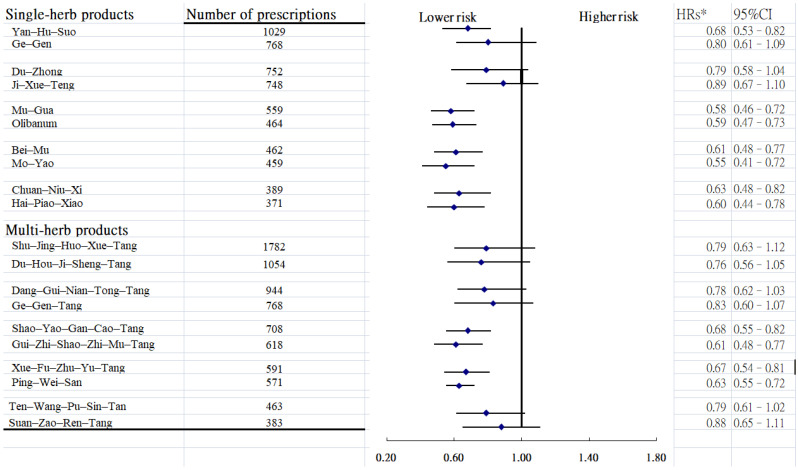
Risk of dental disease with regard to the top ten frequently prescribed single-and multi-herbal products. * Model adjusted for age, gender, urbanization level, monthly income, and CCI.

Among commonly prescribed herbal formulas, 11 prescriptions were found to correlate with a lower risk of dental diseases (Figure 3). Of them, seven were single-herb formulations (Hai-Piao-Xiao, Yan-Hu-Suo, Chuan-Niu-Xi, Mo-Yao, Olibanum, Bei-Mu, and Mu-Gua) and four were multi-herb formulations (Gui-Zhi-Shao-Yao-Zhi-Mu-Tang, Shao-Yao-Gan-Cao-Tang, Xue-Fu-Zhu-Yu-Tang, and Ping-Wei-San).

## 4. Discussion

As a progressive autoimmune disease, RA and accompanying chronic inflammation can wreak havoc on organs throughout the body, particularly the oral cavity [5,24]. Importantly, concurrent dental diseases may impair quality of life in susceptible individuals, thus breeding suicidal ideation or behavior [12]. These repercussions highlight the need for novel therapeutic strategies for oral manifestations. In this longitudinal retrospective cohort study, we observed a 25% reduced risk for dental diseases among those with RA receiving both HMs treatment and Western medicine as compared to those merely receiving Western medicine. Individuals who received the combined HMs treatment for a duration exceeding two years exhibited a more substantial reduction of 68%. This dose-dependent response implied a causal relationship between exposure and response. Although few head-to-head comparisons were made, the positive association between HMs use and reduced dental diseases incidence observed in this study coincides with previous evidence and strengthens our understanding of this issue [25].

Based on stratified analysis by oral manifestations, HMs treatment was found to benefit the prevention of dental diseases, such as stomatitis, periodontitis, pulpitis, and dental caries. However, there was a small, non-significant reduction in the number of gingivitis and oral ulcerations, perhaps due to the small sample size to incur the inflated chance of type II error. Collectively, the positive effect of HMs on the prevention of dental diseases may be tied to the strong anti-inflammatory activity [26,27]. Moreover, several ingredients extracted from herbal products have exhibited antibacterial and wound-healing properties [28]. All of these properties are crucial for gum health [2,29].

One major contribution of the current study is that it puts forward the specific herbal constitutions that may mitigate the risk of dental diseases in patients with RA. Among commonly prescribed single-herb formulations, Mu-Gua and Olibanum showed potential for preventing dental disorders. In agreement with earlier reports [30,31], both herbs are frequently employed to cure arthritis and related symptoms, such as muscle cramps and joint pain. The positive impacts may relate to the respective constituents, such as boswellin in Olibanum and flavonoids in Mu-Gua, both of which exerted potent antioxidant, anti-inflammation, and antibacterial properties [14,15]. One in vitro study reported that boswellic acids effectively inhibited the proliferation of *Aggregatibacter actino mycetemcomitans*, a Gram-negative bacterium that colonizes the human oral cavity and contributes to the pathogenesis of gum diseases [32]. Additionally, flavonoids were found to suppress the growth and adhesion of *Porphyromonas gingivalis* [33]. Likewise, Bei-Mu and Mo-Yao were also significantly associated with a reduced risk of dental disease. In clinical settings, these two herbs have been customarily used to ease muscle soreness. Previous experiments addressed these two herbs had anti-inflammatory and immune regulatory effects in peripheral macrophages by influencing the activation of p38 mitogen-activated protein kinase (MAPK) signaling [34,35]. This process is instrumental in the breakdown of bone matrix proteins, a condition frequently associated with periodontal disease.

We also demonstrated the positive effects of Chuan-Niu-Xi, Yan-Hu-Suo, and Hai-Piao-Xiao on the prevention of dental diseases. A rat fed with the cyasterone, one major substance isolated from Chuan-Niu-Xi, showed lower activity of inducible inflammatory molecules via regulating the nuclear factor kappa B (NF-κB) and MAPK pathways [36]. Activation of MAPK signaling is recognized to induce the expression of transcription factors, such as nuclear factor of activated T-cell cytoplasmic 1 (NFATc1) and AP-1, which in turn promoted osteoclasts differentiation [37]. As to the benefits of the other two medicinal plants, we inferred that several scientific explanations are behind it. An in vitro study showed that berberine, a core component of Yan-Hu-Suo, significantly inhibited the proliferation of oral pathogenic bacteria, especially *Tannerella forsythia*, a Gram-negative bacterium associated with severe periodontitis [38]. Similarly, chitosan, a major deacetylated derivative of Hai-Piao-Xiao, displayed a broad spectrum of antibacterial activityagainst numerous periodontal pathogens, including *Porphyromonas gingivalis*, *Treponema denticola*, and *Prevotella intermedia* [39,40]. Seeing these facts, it is probable that herbal formulations can prevent the onset of dental diseases in a natural manner.

Among commonly prescribed multi-herb formulas to treat RA, those taking Xue-Fu-Zhu-Yu-Tang or Shao-Yao-Gan-Cao-Tang also had a notably lower risk of dental diseases. Modern pharmacological experiments in rodent models suggest these formulas can efficiently abate the levels of plasma cytokines, such as tumor necrosis factor-α, IL-1β and IL-6, by inhibiting the toll-like receptor 4/nuclear factor kappa B pathway [41,42]. This signaling pathway was believed to serve as a primary mechanism linking inflammation and tissue destruction to gum diseases, activated by pathogens like *Porphyromonas gingivalis* [8].

Participants who received Ping-Wei-San or Gui-Zhi-Shao-Yao-Zhi-Mu-Tang also had a decreased risk of sustaining dental diseases. Based on earlier in vivo and in vitro studies, these formulas were reported to alleviate the expression of inflammatory mediators by modulating the phosphatidylinositol 3 -kinase–Akt signaling pathway [43,44], which is an important inflammasome pathway involved in osteoclast differentiation and bone remodeling [37]. As for Dang-Gui-Nian-Tong-Tang, it is a frequently used herbal formula designed to address Qi deficiency in the organs, a common condition associated with RA that affects joint pain. A recent animal study demonstrated that Dang-Gui-Nian-Tong-Tang would mitigate the symptoms of bone erosion and joint destruction, as well as regulate mitochondrial apoptosis by reducing the abundance of prevotella [16]. This bacterium has been noted to quickly pile up in the gut soon after RA emergence [17]. Hence, gut microbiome analysis should be integrated timely into routine RA care to boost treatment efficacy while managing oral hygiene [9].

Despite promising findings, this study has important limitations that must be acknowledged. First, HMs use and disease exposure were identified in accordance with ICD-9-CM codes; however, code errors are inevitable. Accordingly, we only identified those having either RA or dental diseases after at least three outpatient visits reporting consistent diagnoses or one inpatient admission that was recorded. Besides that, Taiwan’s National Health Insurance program randomly samples claims from hospitals, interviews patients, and reviews medical charts to make sure the diagnostic codes are accurate. The association would therefore likely be underestimated rather than overestimated if a misclassification occurred. Second, we could not account for other confounding factors such as tobacco use, alcohol use, physical activity, dietary preferences, social network relationships, or educational level, as all of them were unavailable in the database. Future studies may investigate these untested factors via random assignments across diverse ethnic groups to clarify the generalizability of our findings. Third, data regarding RA severity were unavailable in the studied database, thus likely damaging the applicability of our findings. However, multivariate analysis was employed to consider the impact of synchronous comorbidities assessed by CCI scores. Furthermore, we carried out another sensitivity analysis with the prescription of biological agents as a surrogate variable. All participants were divided into two groups: those who received biological agents for more than six months (group one) or less than six months (group two) after the index date. With control for this surrogate, the benefit of HMs appeared to reduce but remained significant, with the adjusted HRs of 0.83 (95% CI = 0.74–0.95). Hence, we suggest that RA severity did not bias the results of this study. In spite of these concerns, the present work also has several strengths. First, to our knowledge, this is the first study to compare the risk of dental diseases between RA patients who received only conventional Western medicine and those who received the addition of HMs treatment under the same insurance payment system. Second, the large study population allowed for the assessment of infrequent conditions, especially RA. Third, this register-based cohort study greatly benefited from minimization of the selection bias and loss to follow-up. In summary, our findings suggest that adding HMs treatment into conventional Western medicine substantially mitigates sequent risk of dental diseases among RA patients, which can be a reference in designing more appropriate remedies for such groups.

## 5. Conclusions

Findings from this study are helpful to pin down the association of HMs with sequent likelihood of developing dental diseases, demonstrating that combining HMs treatment with conventional Western medicine care of RA greatly decreased risk of dental diseases. Clarification of the dose–response relationship further suggested a causal link between exposure and outcome. Notably, we identified several commonly prescribed herbs that may be associated with a lower risk of dental diseases, thereby providing a foundation for further comprehensive pharmacological experiments targeting the treatment of additional disorders. In terms of clinical implications, rheumatologic clinic practitioners and people living with RA should be aware of the higher risk of dental diseases and remain vigilant by regularly monitoring for early signs through identification of gut microbiota imbalances. At the same time, the administration of appropriate diagnostic tests and provable treatments in RA treatment are urgently needed to abate the chance of developing dental diseases.

## Figures and Tables

**Table 1:** Demographic data and comorbidity comparison of the study subjects.

Variables	All Subjects (*n* = 2359)	Non-HMs Users	HMs Users	*p*
		(*n* = 1711)	(*n* = 648)	
Age, (years)				0.46
≤50	770 (32.6)	547 (32.0)	223 (34.4)	
>50	1589 (67.4)	1164 (68.0)	425 (65.6)	
Mean (SD)	57.3 (13.5)	57.4 (13.6)	57.7 (14.1)	0.61
Gender				<0.01
Female	1484 (62.9)	1035 (60.5)	449 (69.3)	
Male	875 (37.1)	676 (39.5)	199 (30.7)	
Monthly income				0.12
Low	935 (39.6)	694 (40.6)	241 (37.2)	
Median	1370 (58.1)	983 (57.4)	387 (59.7)	
High	54 (2.3)	34 (2.0)	20 (3.1)	
Residential area				0.47
Urban	1110 (47.1)	793 (46.3)	317 (48.9)	
Suburban	386 (16.4)	280 (16.4)	106 (16.4)	
Rural	863 (36.6)	638 (37.3)	225 (34.7)	
CCI	6.1 (9.1)	6.4 (9.4)	5.4 (7.5)	0.02

**Table 2:** Crude and adjusted HRs of dental diseases for RA patients with and without receiving HMs.

Patient Group	Event	PY	Incidence	Crude HRs (95% CI)	Adjusted HRs * (95% CI)
Non-HM users	1012	9463.65	106.94	1	1
HM users	375	4156.79	90.21	0.84 (0.75–0.94)	0.75 (0.66–0.86)
Use frequency_Q1	344	3587.45	95.89	0.90 (0.78–0.98)	0.81 (0.71–0.91)
Use frequency_Q2	24	383.45	62.59	0.59 (0.39–0.81)	0.48 (0.33–0.72)
Use frequency_Q3	7	185.59	37.72	0.35 (0.17–0.70)	0.32 (0.15–0.68)

Incidence rate is per 1000 PY. * Model adjusted for age, gender, urbanization level, monthly income, and comorbidities. Q1: HMs use for less than one year; Q2: HMs use for 1–2 years: Q3: HMs use for 2 years or more.

**Table 3:** Risk for different dental diseases in relation to HMs use or not.

Variables	Participants *n* (%)	Crude HRs (95% CI)	Adjusted HRs * (95% CI)
Dental caries (*n* = 427)			
Non-HMs users	321 (75.2)	1	1
HMs users	106 (24.8)	0.77 (0.61–0.85)	0.75 (0.61–0.94)
Pulpitis (*n* = 149)			
Non-HMs users	119 (80.0)	1	1
HMs users	30 (20.0)	0.76 (0.57–1.04)	0.65 (0.43–0.98)
Gingivitis (*n* = 61)			
Non-HMs users	40 (65.6)	1	1
HMs users	21 (34.4)	0.90 (0.61–1.21)	0.86 (0.57–1.20)
Periodontitis (*n* = 537)			
Non-HMs users	388 (72.3)	1	1
HMs users	149 (27.7)	0.74 (0.60–0.92)	0.74 (0.60–0.91)
Oral ulceration (*n* = 78)			
Non-HMs users	52 (66.7)	1	1
HMs users	26 (33.3)	0.92 (0.67–1.35)	0.94 (0.66–1.38)
Stomatitis (*n* = 135)			
Non-HMs users	93 (68.9)	1	1
HMs users	42 (31.1)	0.69 (0.42–0.92)	0.63 (0.42–0.94)

Incidence rate is per 1000 PY. * Model adjusted for age, gender, urbanization level, monthly income, and CCI.

## Data Availability

The data presented in this study are available on request from the corresponding authors.
